# Haken-Entropy-Based Analysis of the Synergy Among Financial Support, Technological Innovation, and Industrial Upgrading

**DOI:** 10.3390/e28040465

**Published:** 2026-04-17

**Authors:** Yue Zhang, Jinchuan Ke, Jingqi He

**Affiliations:** School of Economics & Management, Beijing Jiaotong University, Beijing 100044, China; 22110176@bjtu.edu.cn (Y.Z.); 25110257@bjtu.edu.cn (J.H.)

**Keywords:** financial support, technological innovation, synergy, industrial upgrading

## Abstract

This study reveals the internal mechanism of the synergetic evolution of financial support, technological innovation, and industrial upgrading from the perspective of system synergy. It aims to provide a theoretical basis and reference for promoting benign interactions among these elements, thereby driving high-quality economic development. During the research process, an evaluation indicator system was constructed based on China’s industrial development data, utilizing the entropy method to determine indicator weights and the Haken model to analyze synergy effects. In a methodological innovation, this study identifies the system’s order parameters to derive the potential function. Through this approach, it systematically analyzes the dynamic evolution characteristics and synergetic mechanisms of the composite system. The research results indicate that the three systems have formed a mutually promoting and closely coupled compound synergetic mechanism, rather than following a single linear transmission path. The overall synergy level presents a medium-to-low development trend, following an asymmetric U-shaped evolution trajectory that first decreases and then slowly recovers. Furthermore, the degree of synergy exhibits an inverse relationship with the volatility of the subsystems, suggesting that the stability of synergy is highly susceptible to external forces and remains in a state of dynamic flux.

## 1. Introduction

In the context of global economic integration, structural contradictions are prevalent across many nations, manifesting as a disconnect between financial systems and industrial requirements. The manufacturing sector remains excessively dependent on traditional labor-intensive models; however, the lag in technological development renders these current development models unable to adapt to the imperatives of economic transition. This reliance on low-efficiency production modes inherently restricts the capacity for high-value-added growth, which, when coupled with severe financing constraints, exacerbates the urgency for structural reconfiguration. Consequently, under the dual pressure of capital scarcity and technical stagnation, it is imperative to intensify innovation investment and accelerate the adjustment of the economic structure [1,2].

The relationship between financial support (FS) and technological innovation (TI) is mutually reinforcing, necessitating a high degree of synergy. Financial development provides the requisite capital for technological advancement, while science and technology establish the material foundation for the evolution of financial markets. Specifically, technological innovation empowers traditional financial markets, providing sustained innovation momentum for their evolution. Simultaneously, the financial market plays an irreplaceable role in optimizing resource allocation and facilitating the commercial realization of technological achievements [3]. Driven by emerging technologies such as big data, artificial intelligence, cloud computing, and blockchain, technological innovation has catalyzed a series of innovative financial products and services. These advancements provide efficient financing channels for the real economy. By enhancing the accessibility of financial services, technological innovation facilitates the optimization and upgrading of industrial structures (IU), thereby promoting coordinated progress between financial development and industrial modernization [4]. Conversely, the excessive integration and unchecked expansion of financial markets and technological innovation can precipitate structural imbalances. This is often driven by the decoupling of financial capital from real industrial needs or the formation of speculative bubbles through over-leveraged technology investments, which leads to significant capital misallocation. Such phenomena cause technological development to deviate from actual industrial requirements, diminishing the precision of financial services in supporting industrial upgrading and weakening the effectiveness of technological innovation in optimizing industrial structures, ultimately eroding the capacity to drive industrial transformation.

Regarding the co-evolutionary relationship among financial support (FS), technological innovation (TI), and industrial upgrading (IU), complex system theory has facilitated a transition from qualitative description to quantitative analysis, giving rise to scientific analytical methods such as coupling coordination models, system dynamics, and econometric models. Given that the evolution of economic systems is typically characterized by non-linearity, self-organization, and dynamic progression, the Haken model—built upon self-organization theory—is particularly suitable for characterizing the actual state of synergy among FS, TI, and IU. This model breaks the traditional linear causal research paradigm. By identifying order parameters, it maps the internal dynamic characteristics of the composite system as it transitions from disorder to order. Within this hierarchical framework, the potential function describes the steady-state characteristics of the system at different equilibrium points, while the synergy degree model provides a quantitative evaluation of the overall coordination level among subsystems. These elements are further linked by the analysis of order parameter volatility, which effectively identifies the inhibitory effects of external shocks on system orderliness. Consequently, the objective of this study is to use the Haken model to quantitatively identify the synergy among FS-TI-IU, reveal its synergistic mechanism, and provide a theoretical basis for promoting the coordinated development of industries.

## 2. Literature Review

Technological innovation serves as the primary engine of industrial upgrading, manifesting through distinct regional and global dynamics across environmental and socio-economic dimensions. In the context of green transformation, research highlights a strong synergy between innovation and structural evolution. Regarding OECD nations, Marra et al. (2024) found that R&D-driven progress reduces energy intensity, whereas industrial servitization promotes green innovation to improve environmental quality [5]. Similarly, in China, Dai et al. (2022) used a PVAR model to confirm that technological innovation drives emission reductions by catalyzing industrial upgrades [6]. Ma et al. (2023) further noted that digital transformation and innovation in China not only reduce PM2.5 concentrations but also indirectly facilitate the spatial optimization of industrial structures [7]. Beyond environmental impacts, technological advancement reshapes production efficiency and factor allocation across diverse economies. Analyzing African production systems, Mensah et al. (2023) argued that technological catch-up and cross-sectoral resource reallocation, rather than original innovation, are the primary drivers of efficiency gains [8]. On a broader scale, Guo et al. (2022) found that capital deepening and productivity growth trigger factor reallocation, with labor-enhancing technologies primarily driving skill premiums [9]. Xu (2022) employed a multi-national general equilibrium framework to analyze these premiums [10]. The study confirms that technological progress facilitates a transition toward skill-intensive sectors, which acts as a major driver for the global rise in skill premiums. This shift is echoed in Brazil, where Gabriel et al. (2025) concluded that demand growth alone is insufficient for upgrading; instead, technological innovation is essential to strengthen the correlations between manufacturing and service sectors [11]. However, the transition also presents significant challenges for labor markets and structural equity. Boddin and Kroeger (2025) found that while skill-oriented technical advancement stimulates job growth in Germany, conventional structural changes remain responsible for the majority of manufacturing employment losses [12]. Furthermore, Cauvel and Pacitti (2022) warned that structural factors such as financialization and technological change may erode workers’ bargaining power and alter income distribution patterns during the upgrading process [13]. Collectively, these studies indicate that while technological innovation is the fundamental catalyst for industrial upgrading, its impact varies by developmental stage. While advanced economies leverage innovation for green transitions and skill-intensive growth, developing regions often rely on technological catch-up and resource reallocation, necessitating a balanced approach between efficiency gains and social stability.

In the domain of financial support for technological innovation, recent research from 2020 to 2025 reveals an analytical shift from traditional funding mechanisms toward specialized digital and green financial tools. This evolution reflects an increasing focus on how modern fintech ecosystems precisely target environmental objectives. Meng et al. (2022) demonstrated that digital finance elevates regional green innovation by mitigating financing hurdles and fostering information sharing [14]. He et al. (2024) further identified a U-shaped relationship between fintech development and green advancements within the banking sector [15]. From an industrial perspective, Bu et al. (2025) utilized machine learning to show that fintech strengthens the innovation capacity of manufacturing firms by expanding R&D resources [16]. In contrast, adopting a geographical lens, Li et al. (2025) utilized urban data to show that fintech fosters innovation primarily by attracting foreign direct investment [17]. Complementing these macro-trends, Hunjra (2025) provided micro-evidence showing that green finance successfully reshapes corporate capital allocation toward sustainable technological progress [18]. Regarding financial support for industrial upgrading, the literature emphasizes the transition from structural growth to high-quality, sustainable transformation. Zhu (2022) found that green finance facilitates the modernization of industrial structures by leveraging corporate innovation as a catalyst for green growth [19]. In rural and emerging contexts, Garg et al. (2025) confirmed that expanding banking access promotes non-agricultural entrepreneurship and labor migration [20]. However, the success of such transitions is often contingent upon institutional quality. Ali et al. (2023) observed that financial support is less effective at upgrading resource-dependent regions if it is disconnected from sustainable management frameworks [21], while Quist (2022) argued that the governance of the financial system determines the execution of industrial transformation strategies [22]. Historical evidence from Basco and Tang (2020) suggests that the synergy between finance and infrastructure guides the shift toward service-oriented economies, though long-term outcomes remain market-dependent [23]. Ultimately, Moussir and Chatri (2020) reinforced the connection between financial systems and structural change, highlighting that financial reform and human capital are essential drivers for fostering emerging industries and enhancing total factor productivity [24].

To enhance clarity, this section examines the synergy between financial support, technological innovation, and industrial structures, transitioning from macro-institutional frameworks to the specific mechanisms of digital and green finance, and concluding with regional heterogeneous applications. From a macro-regulatory perspective, financial oversight provides the institutional scaffolding necessary for structural modernization. Li and Hou (2025) demonstrate that regional financial regulatory spending correlates positively with enterprise patenting, suggesting that robust regulation prevents capital diversion and directs resources toward research and development [25]. Recent scholarship highlights a dual-pathway interaction where digital and green finance function as mutual catalysts for industrial evolution. Hu et al. (2025) observe that technological innovation moderates the relationship between digital finance and industrial upgrading, effectively reducing energy footprints through this triple-subsystem linkage [26]. Complementing this, Zhang and Ai (2025) posit that digital finance optimizes resource allocation, thereby bolstering industrial ecological resilience and technological progress [27]. Gao et al. (2022) emphasize that green innovation, guided by targeted financial resources, reduces emissions primarily by mediating structural transitions [28]. Similarly, Zhao et al. (2025) argue that green credit instruments regulate the expansion of carbon finance, which in turn facilitates structural optimization [29]. Within the context of open economies, Li et al. (2025) confirm that digital inclusive finance optimizes trade performance through the industrial upgrading-technological innovation nexus [30]. Soltani (2024) synthesizes these perspectives, identifying the synergy between digitalization and green financial instruments as a fundamental engine for achieving carbon neutrality [31]. The effectiveness of these transmission mechanisms varies across regional contexts, characterized by distinct spatiotemporal patterns and developmental priorities. While Guo et al. (2024) show that integrating digital and real economies improves green productivity [32], and Wang et al. (2023) highlight how fiscal decentralization leverages energy efficiency through policy intervention [33], other studies underscore the broader socio-economic impacts of these synergies. For instance, both Endale et al. (2024) and Nie et al. (2022) explore how the coupling of financial support and structural transformation manifests across different environments; the former focuses on agricultural modernization as a tool for poverty reduction [34], while the latter identifies the spatiotemporal evolution of green-industrial coordination [35]. Finally, Wang et al. (2021) empirically confirm that regional green development in pilot zones is driven precisely by the “innovation-upgrading” linkage [36].

Research methods examining the relationship between financial support, technological innovation, and industrial upgrading can be categorized into system synergy measurement, spatiotemporal analysis, and causal inference. These include coupling coordination degree models, spatial econometric VAR models, panel data regression models, and complex network models. The coupling coordination degree measures the degree of benign interaction and mutual influence between two or more subsystems. For instance, Wang and Dong (2024) employed coupling models to measure the interactive effects between digital finance and green technology innovation [37]. Liu and Dong (2021) focused on the impact of technological innovation on urban green economy efficiency [38]. To capture the evolutionary characteristics of systems across geography, spatial econometric analysis is often utilized. Wang et al. (2024) explored the spatiotemporal coupling relationship between regional development factors [39]. Wu and Xie (2025) revealed the dynamic evolution patterns—defined as the trajectory and characteristics of system state changes over time—of the digital economy and ecological efficiency [40]. Lv et al. (2024) applied similar methods to the “water–energy–food” nexus system [41]. While these methods effectively depict the external state of system collaboration, they often lack an exploration of internal dynamic mechanisms. Specifically, they fail to clarify the micro-level feedback loops and transmission pathways that drive system transitions, often treating the internal interaction as a “black box”. Regarding causal relationship testing, panel data regression models, such as Ordinary Least Squares (OLS), are frequently used to analyze linear influences. Ning and Shun (2025) and Shao et al. (2025) utilized enterprise-level micro-data to investigate the effects of financial policies [42,43]. Their findings suggest that digital and technology finance policies significantly promote innovation performance. Wang et al. (2025) further explored the causal impact of new-era entrepreneurship on manufacturing green innovation efficiency [44]. Although these regression methods excel at quantifying policy effects, they struggle to fully characterize the complex nonlinear interaction processes among the internal elements of a composite system. In recent years, analysis methods based on complex system theory and complex networks have received increasing attention. Unlike traditional linear models, complex network analysis can map topological structures and identify systemic vulnerabilities, making it superior for handling non-linear dependencies and high-dimensional system problems. Gao et al. (2024) analyzed the impact of technology finance policies on systemic risks by constructing a bank-enterprise network model [45]. Chen et al. (2025) explored how blockchain financial technology transformation mitigates supply chain disruption risks and enhances overall system stability [46].

In summary, current research on the interaction mechanisms among financial support, technological innovation, and industrial upgrading focuses primarily on single or two-way influence paths, lacking in-depth exploration of the system’s overall synergetic effects. Methodologically, existing studies rely heavily on traditional statistical regression and econometric models, which emphasize linear correlations but often fail to characterize nonlinear interactions and dynamic evolutionary processes. To address these limitations and provide a more integrated perspective, this study, by extending the theoretical foundations of existing research, proposes a multidimensional framework focusing on the following three aspects: First, this study conceptualizes financial support, technological innovation, and industrial upgrading as a composite system, reflecting the complexity and systematic nature of the modern economy. By emphasizing a system dynamics approach, this framework shifts the focus toward the synergy and nonlinear patterns inherent within the system, thereby moving beyond the constraints of traditional linear analysis. Second, the Haken model is introduced to quantitatively characterize and compare the co-evolutionary paths of the three subsystems: financial support and technological innovation (FS-TI), financial support and industrial upgrading (FS-IU), and technological innovation and industrial upgrading (TI-IU). By streamlining the identification of order parameters and the construction of potential functions to solve motion equations, this model effectively captures the internal driving forces of system self-organization. Finally, from the perspective of dynamic evolution, this study analyzes the response mechanisms and stability differences in the system’s synergetic state under external shocks. This approach allows for a more comprehensive revelation of the evolutionary laws governing synergetic effects and provides nuanced policy implications—such as optimizing the allocation of financial resources to stabilize innovation-driven growth or tailoring industrial policies to specific evolutionary stages—thereby enhancing the practical relevance and persuasiveness of the findings.

## 3. Model Construction

The interaction between financial support (FS), technological innovation (TI), and industrial upgrading (IU) is not a simple linear accumulation but a complex network of internal connections driven by capital allocation, technology diffusion, and industrial adjustment. While traditional econometric methods effectively identify causal relationships between variables, they often struggle to capture the underlying synergy characteristics among these three components. The Haken model addresses this limitation by identifying the “order parameters” that govern the evolution of complex systems. Its unique advantage lies in its ability to simplify multi-dimensional interactions into manageable equations of motion, allowing researchers to determine whether the non-linear feedback loops between FS, TI, and IU—such as capital fueling R&D which then accelerates structural shifts—result in a self-organized, synergistic state [47,48].

### 3.1. Haken Model

#### 3.1.1. Order Parameter Equation

The Haken model is built upon the principle of the motion equation to describe how a system’s state evolves. In this framework, the state effect q(t) at any given time t is influenced by internal dynamics and a decaying external force, represented as $F\left(t\right)={ae}^{-\delta t}$ [49]. In the context of industrial systems, this external force might represent a singular policy intervention or a market shock that gradually diminishes.

(1) $$\dot{q}\left(t\right)=\gamma q+F\left(t\right)$$

(2) $$q\left(t\right)=\frac{a}{\gamma -\delta }\left(e^{-\delta t}-e^{-\gamma t}\right)$$

where α is a constant representing the initial magnitude of the external influence. The parameters γ and δ serve as damping coefficients for the system and the external force $F\left(t\right)$, respectively. Specifically, γ reflects the system’s internal stability and its speed in responding to changes, while δ determines how quickly the external stimulus loses its impact over time. By comparing these coefficients across the FS, TI, and IU subsystems, the model identifies which factor evolves most slowly and thus acts as the dominant “order parameter” directing the entire system’s development. For two interacting subsystems $q_{1}\left(t\right)$ and $q_{2}\left(t\right)$, for example, where financial investment $q_{1}\left(t\right)$ provides the liquidity necessary for technological breakthroughs $q_{2}\left(t\right)$, their interactive motion can be expressed as follows [50]:

(3) $$\frac{dq_{1}(t)}{dt}=-\gamma_{1}q_{1}(t)-aq_{1}(t)q_{2}(t)$$

(4) $$\frac{dq_{2}(t)}{dt}=-\gamma_{2}q_{2}(t)+bq_{1}^{2}(t)$$

where *a* and *b* are variables reflecting the interaction intensity of $q_{1}\left(t\right)$ and $q_{2}\left(t\right)$. If the damping condition assumption is to be satisfied, the damping coefficient of the system must be greater than the damping coefficient of the external force, that is, $\left|\gamma_{2}\right|>\left|\gamma_{1}\right|$ and $\gamma_{2}>0$, which indicate the state variable is a fast variable with rapid decay.

Under the condition $\left|\gamma_{2}\right|\gg \left|\gamma_{1}\right|$, the subsystem *q*_2_ exhibits stable and damped behavior, reacting much faster than the internal force *q*_1_. Consequently, *q*_2_ can be assumed to reach a quasi-steady state relative to *q*_1_, justifying the assumption $\dot{q_{2}}$ = 0. Let $\dot{q_{2}}$ = 0, then.

(5) $$q_{2}(t)=\frac{b}{\gamma_{2}}q_{1}^{2}(t)$$

(6) $$\frac{dq_{1}(t)}{dt}=-\gamma_{1}q_{1}(t)-\frac{ab}{\gamma_{2}}q_{1}^{3}(t)$$

To further analyze the dynamics of *q*_1_ the motion equations can be changed to

(7) $$q_{1}(t)=(1-\gamma_{1})q_{1}(t-1)-aq_{1}(t-1)q_{2}(t-1)$$

(8) $$q_{2}(t)=(1-\gamma_{2})q_{2}(t-1)+bq_{1}^{2}(t-1)$$

#### 3.1.2. Potential Function

One can examine the system’s energy landscape through its potential function. This function describes the transformation of the system from one form of energy to another and can be utilized to evaluate the system’s stability state comprehensively. Specifically, the system potential function is obtained by integrating the negative derivative of $\frac{dq_{1}(t)}{dt}$, with respect to time:

(9) $$v(q_{1})=\frac{1}{2}\gamma_{1}q_{1}^{2}(t)+\frac{ab}{4\gamma_{2}}q_{1}^{4}(t)$$

**Figure 1:**
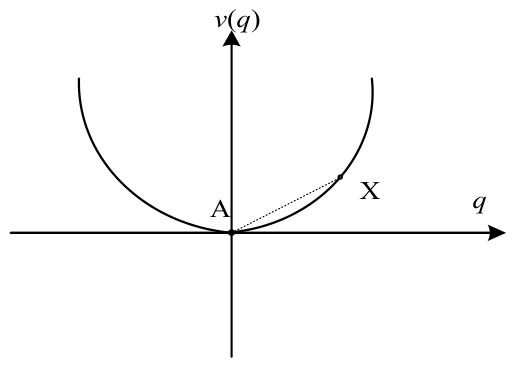
A potential function of the unique solution.

The qualitative stability of the system and the derivation of its equilibrium solutions are fundamentally governed by the sign of the coefficient product ($a*b*\gamma_{1}*\gamma_{2})$. If ($a*b*\gamma_{1}*\gamma_{2})$ > 0, the potential function possesses only one stable point A, yielding a unique solution $q_{1}^{*}=0$. In this state, the condition of any point X in the system is determined by its distance from the stable point A equilibrium, as illustrated in Figure 1. Conversely, if ($a*b*\gamma_{1}*\gamma_{2}$) < 0, the potential function equation admits three distinct solutions:

$$q_{1}^{*}=0;q_{1}^{**}=\sqrt{\left|\frac{\gamma_{1}\gamma_{2}}{ab}\right|};q_{1}^{***}=-\sqrt{\left|\frac{\gamma_{1}\gamma_{2}}{ab}\right|}$$

**Figure 2:**
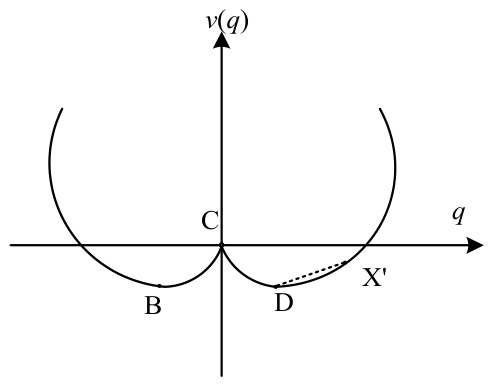
A potential function with a non-unique solution.

At this stage, while $q_{1}^{*}(t)$ represents a solution to the equilibrium equation, it exhibits inherent instability. Driven by shifts in the system’s control parameters that alter the underlying potential landscape, the stable state point C undergoes a displacement mechanism. As illustrated in Figure 2, it causes the system to transition away from its precarious state and eventually settle into one of the two final equilibrium points B and D which represents $q_{1}^{**}(t)$ and $q_{1}^{***}\left(t\right)$, respectively. Within this framework, X′ is defined as a transient state point that characterizes the system’s instantaneous configuration. The relative stability of X′ is determined by its distance from the equilibrium points B or D.

#### 3.1.3. Degree of Synergy

In the potential function, the distance between any point X′ and the equilibrium point reflects the system’s proximity to a steady state and its potential for dynamic recovery. Consequently, a state evaluation function can be established as follows:

(10) $$d(t)=\sqrt{{\left[q_{x^{\prime }}(t)-q_{1}^{*}(t)\right]}^{2}+{\left[v\left(q_{X^{\prime }}\right)-v\left(q_{1}^{*}\right)\right]}^{2}}$$

The value of d reflects the synergy among financial support, technological innovation, and industrial upgrading. A larger d value indicates that the system is farther away from the stable state, signifying a lower level of systemic synergy. If the order parameter is a positive variable, the necessary condition for the system to transition from point X′ back to a stable equilibrium is $q_{X^{\prime }}<q_{1}^{*}$. This condition implies that the state remains within the basin of attraction defined by the potential function, where the restorative forces dominate. As $q_{X^{\prime }}$ gradually approaches $q_{1}^{*}$, the system undergoes a dynamic convergence process characterized by diminishing fluctuations and the alignment of sectoral growth rates, which facilitates a structured return to a stable state.

Based on normalized indicators, the degree of synergy (*S*) can be defined to represent the interactive efficiency and overall coherence of the composite system.

(11) S(t)=1−d(t)

A higher *S* value signifies a superior level of systemic coordination. Drawing upon the classification framework of Zhu and Yang (2023) [51], the degree of synergy is categorized into three distinct levels: low-level synergy (0 < *S* ≤ 0.40), moderate synergy (0.4 < *S* ≤ 0.7), and high-level synergy (0.7 < *S* ≤ 1.0). These evaluation criteria are summarized in Table 1.

While these synergy levels provide a macro-level assessment of coordination, they do not pinpoint the specific internal drivers of systemic disparity. To complement this evaluation and identify structural imbalances during the co-evolution process, this study adopts the Theil’s Entropy Measure. By utilizing the concept of information entropy to calculate disparities in development, this model facilitates a more granular assessment of the balanced development among the three subsystems: financial support, technological innovation, and industrial upgrading.

(12) $$T_{t}=\sum_{i=1}^{n}\left[\frac{l_{it}}{Ave(l_{it})}\times \ln\left(\frac{l_{it}}{Ave(l_{it})}\right)\right]$$

where $T_{t}$ represents the Theil index in year *t*, indicating development imbalances among the three subsystems; *n* is the number of subsystems; $l_{it}$ represents the development index value of subsystem *i* in year *t*; and $Ave(l_{it})$ is the average development value of the three subsystems in year *t*.

## 4. Empirical Analysis of the Synergistic Effects Among FS, TI, and IU

### 4.1. Hypothesis

A composite system is defined as an integrated structure in which multiple subsystems interact and co-evolve to produce systemic functions that transcend the simple sum of their individual components. Within this framework, financial support, technological innovation, and industrial upgrading do not merely exhibit a linear superposition relationship, but rather achieve interaction through resource allocation and functional synergy. Financial development optimizes the structure and efficiency of capital supply, which catalyzes technological innovation inputs and facilitates the transformation of the industrial structure. This process establishes a circular feedback loop: the industrial growth and market opportunities arising from technological innovation provide more robust investment targets for financial assets, while the emergence of new products and processes enhances the total factor productivity of traditional industries, driving the industrial structure toward more advanced levels. Simultaneously, the industrial agglomeration formed during the process of industrial upgrading fosters a conducive environment for knowledge spillovers, which reduces innovation costs and further stimulates technological progress. Furthermore, the increasing complexity of economic activities inherent in industrial upgrading expands the demand for financial services beyond basic deposits and loans to diversified businesses such as venture capital, mergers and acquisitions, and asset securitization, thereby compelling the financial sector to pursue structural development and product innovation. This feedback chain, based on element cycling, enables FS, TI, and IU to form mutually reinforcing and co-evolving characteristics throughout the developmental process. Thus, the following hypothesis is proposed.

**Hypothesis** **1.** *There is a synergistic effect between financial support, technological innovation, and industrial upgrading.*

According to synergy theory, the degree of synergy within the FS-TI-IU composite system represents its overall orderliness and stability. Excessive volatility exacerbates market uncertainty. These fluctuations inhibit long-term investment and technological innovation, thereby disrupting industrial operations and resulting in decreased synergy. Conversely, low volatility ensures stable system operation, which is conducive to capital allocation, technological accumulation, and industrial iteration. Given that systemic stability is a prerequisite for the harmonious interaction of these components, this study proposes the following:

**Hypothesis** **2.** *The volatility of the composite system is negatively correlated with its overall synergy.*

### 4.2. Establishment of Indicator System

#### 4.2.1. Indicator Selection

(1) Index of Financial Support

To empirically test the relationship between system stability and synergy as posited in Hypothesis 2, this study draws on the work of Li et al. (2025), who emphasize the structural determinants of financial system order [52]. Based on their theoretical framework and the developmental characteristics of China’s financial system, a financial support indicator system is constructed. This system is analyzed through several distinct dimensions. These include capital scale, resource allocation, operational efficiency, and green transformation.

X11: Financial industry revenue growth. This indicator measures capital scale and reflects the developmental progress of the financial sector.

X12: Loans for high-tech and industrial upgrading. This metric represents resource allocation and quantifies the credit support provided to the industrial economy.

X13: Transaction amount of electronic banking business. This metric reflects the electronic banking service usage rate; as the increased adoption of digital channels streamlines financial intermediation and enhances capital mobility, it serves as a critical indicator for measuring the operational efficiency of the financial system and the breadth of technological coverage.

X14: Green credit quota. Complementing this focus on digital infrastructure, green credit quota captures the sustainability aspect of financial development. This indicator represents the ratio of green loan amount to total loan amount of banking institutions. It depicts the allocation of financial resources to low-carbon industries and eco-friendly sectors, indicating the extent of financial support provided for industrial upgrading [53].

(2) Index of Technological Innovation

Following the research framework established by Zhang and Ni (2022), this study constructs an evaluation system that systematically bridges the dimensions of innovation input and output [54]. The selected indicators are structured to reflect a logical progression from resource mobilization to market realization, emphasizing the synergistic relationship between investment and tangible results.

X21: The full-time equivalent of R&D personnel, which quantifies the concentration of specialized intellectual labor, a factor that fundamentally determines the depth and sustainability of technological innovation activities [55].

X22: Human capital augmented by R&D investment in high-tech products, representing the targeted financial commitment and resource allocation intensity required for industrial modernization.

X23: The number of authorized patents, which serves as a metric for the proliferation of high-value originality and breakthroughs in core technological fields.

X24: The sales revenue from high-tech products, which evaluates the market-oriented transformation of innovation achievements and their capacity to generate tangible economic value within the industrial chain.

(3) Index of Industrial Upgrading

Industrial structural upgrading represents a pivotal phase in economic development, characterized by the optimization of factor allocation and the systematic progression of industrial hierarchies, which serves as a fundamental driver of sustainable economic growth and industrial modernization. To comprehensively capture this transition, this study evaluates the industrial structure subsystem across the dimensions of labor structure, industrial sophistication, industrial hierarchy, and carbon efficiency. These dimensions are selected because they collectively reflect the core components of industrial modernization: human capital mobility, technological complexity, structural maturity, and environmental sustainability.

X31: Proportion of employment in the tertiary industry, indicating the transition of the labor force from traditional production sectors to high-value-added service sectors, thereby representing the upgrading of human capital within the industrial framework.

X32: Ratio of tertiary added value to that of the secondary industry. This study adopts this ratio as a measurement indicator, indicating the service-oriented transition and industrial advancement [56].

X33: Industrial structure hierarchy index, which reflects the systematic upgrading of the industrial framework. Drawing on the research approach of Yu and Wang (2021), this study utilizes their multi-level weighting method to capture the dynamic evolution across all economic sectors, thereby providing a more comprehensive measure of structural sophistication than single-sector ratios [57]. Specifically, this study uses the weighted GDP proportions from primary, secondary, and tertiary industries. The specific formula is:

(13) $$W=\sum_{i=1}^{3}\left(y_{i}\times i\right),\;i=1,\;2,\;3$$

where $y_{i}$ represents the proportion of the $GDP_{i}$ added value of the *i*-th industry; the weights 1, 2, and 3 are assigned to the primary, secondary, and tertiary industries, respectively, to represent their increasing levels of structural hierarchy.

X34: Unit *GDP* carbon emission reduction. To evaluate the environmental efficiency of industrial upgrading, this study draws on the framework proposed by Chang et al. (2023), which used the carbon emission reduction per unit of to measure the efficiency of industrial low-carbon transformation [58]. The formula is

(14) $$CI(t)=\frac{\Delta CE(t)}{GDP(t)}$$

where ΔCE(t) denotes the amount of carbon dioxide emission reduction in *t* year.

#### 4.2.2. Determination of Indicator Weights

The entropy weight method is employed to construct a comprehensive index for the indicator sub-systems, specifically targeting dimensions such as resource utilization efficiency and environmental carrying capacity. The methodology entails the normalization of sub-indicators, the determination of information entropy, and the application of objective weighting. The resulting composite value serves as the standardized scoring criterion for the evaluation indicators. The specific computational steps are as follows:

(1) Normalization

The raw data are normalized to eliminate dimensional discrepancies among variables by using the Min–Max method to ensure all indicators are comparable within the range of [0,1]:

(15) $$x_{ij}^{\prime }=\frac{x_{ij}-\min\left(x_{j}\right)}{\max\left(x_{j}\right)-\min\left(x_{j}\right)}$$

(2) Calculation of Indicator Proportion

The proportion of the *i*-th object under the *j*-th indicator is calculated as

(16) $$P_{ij}=\frac{x_{ij}^{\prime }}{\sum_{i=1}^{m}x_{ij}^{\prime }}$$

(3) Calculation of Information Entropy

The information entropy value $e_{j}$ for the *j*-th indicator is determined based on the distribution among *m* samples as

(17) $$e_{j}=-\frac{1}{ln\;(m)}\sum_{i=1}^{m}\left(P_{ij}\ln P_{ij}\right)$$

(4) Determination of the Weights

The weight $w_{j}$ for each indicator among *m* indicators is determined based on the variation coefficient:

(18) $$w_{j}=\frac{1-e_{j}}{\sum_{j=1}^{m}(1-e_{j})}$$

(5) The Comprehensive Evaluation Score

The comprehensive evaluation score $U_{i}$ for each object is obtained by calculating the weighted sum of all normalized sub-indicators.

(19) $$U_{i}=\sum_{j=1}^{m}w_{j}x_{ij}^{\prime }$$

In the construction of an evaluation indicator system encompassing financial support, technological innovation, and industrial upgrading, the precise determination of index weights is paramount for integrating these distinct dimensions. To achieve this, the original data undergo dimensionless processing using the entropy weight method, a procedure that eliminates the influence of disparate units and scales, thereby ensuring the objectivity and comparability of the assessment. This systematic processing quantifies the relative importance of each indicator, forming the logical basis for the weight distribution presented in Table 2. These weights are subsequently utilized for the calculation of the comprehensive value of each subsystem, effectively synthesizing the three pillars into a coherent evaluation framework. By establishing this weighted structure, the methodology clarifies the internal synergy between the subsystems and provides a rigorous quantitative foundation for the final assessment of the integrated indicator system.

### 4.3. Data Sampling

During the data collection stage, it was observed that specific provincial-level indicators exhibited extremely minimal numerical values, primarily due to localized economic disparities and data sparsity in certain administrative divisions. To mitigate the potentially distorting effects of these small indicator values on the analytical results, this study aggregates data into nine distinct geographic regions—eastern, western, southern, northern, central, northeastern, northwestern, southeastern, and southwestern China—covering a time series from 2011 to 2024. This methodological choice is strategically intended to reduce data noise and enhance sample robustness, ensuring the dataset satisfies the rigorous damping condition assumptions of the Haken model, which in turn facilitates a more precise capture of the system’s co-evolutionary dynamics.

The empirical framework utilizes a multi-dimensional dataset synthesized from several authoritative sources. The *China Statistical Yearbook* and *China Urban Statistical Yearbook* provide the foundational socio-economic and industrial metrics, while the *China Financial Statistical Yearbook* and the *CNRDS China Research Data Service Platform* supply specialized data regarding financial intermediation and innovation outputs. Additionally, the *China Economic Net* and *EPS* databases serve as supplementary resources for cross-referencing and longitudinal validation. To eliminate dimensional discrepancies and heteroscedasticity between variables, all data underwent rigorous standardization prior to the empirical analysis.

**Figure 3:**
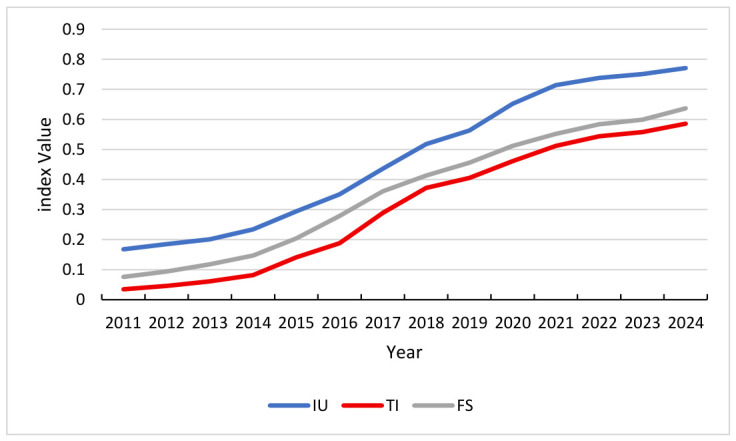
Index value of FS, TI, and IU.

Viewed holistically, the indices for Financial Support, Technological Innovation, and Industrial Upgrading have exhibited a sustained and consistent annual upward trajectory in Figure 3. This steady advancement reflects the progressive optimization of China’s industrial structure and the strengthening of financial and innovation-driven mechanisms under various national strategic frameworks. Specifically, Industrial Upgrading maintains a predominant position in the developmental hierarchy, while Technological Innovation and Financial Support demonstrate accelerated momentum during critical policy transition periods—such as the shifts between Five-Year Plans—where the implementation of targeted subsidies and structural reforms serves as a catalyst for rapid growth. The synchronized elevation of these indices establishes a necessary foundation for system synergies; however, the divergent growth velocities observed across different stages emerge as the primary drivers of system fluctuations, thereby fundamentally shaping the dynamic evolution of the composite system’s synergy level.

### 4.4. Analysis of Synergy Effects

#### 4.4.1. Equation and Parameter Test

(1) Parameter Equation

Financial support, technological innovation, and industrial upgrading constitute a complex composite system. To elucidate their synergistic mechanisms, three bilateral subsystem models (FS-TI, FS-IU, and TI-IU) were constructed based on Haken’s Model of synergetics. This model posits that the macro-evolution of a complex system is governed by “order parameters,” which are slow variables that emerge from the collective interaction of components and dominate the system’s long-term behavior.

The model construction follows a systematic identification process. One variable is designated as the slow variable, representing the core driver with a stable, foundational influence, while the other is treated as a fast variable, which adapts more rapidly to systemic fluctuations. Evolution equations are then established and tested to determine which variable acts as the order parameter, as detailed in Table 3. According to the regression results, the F-statistics for the FS-TI and FS-IU systems are significant at the 0.01 confidence level, with corresponding *p*-values below 0.001. In contrast, the TI-IU system yields an F-statistic of 2.63, which meets the 0.1 significance threshold; notably, its *p*-value of 0.032 further confirms that the model is significant at the 0.05 level. Crucially, these results validate that the model’s identified dynamic evolutionary relationships are statistically reliable, ensuring the identified pathways of influence are not due to random fluctuations.

In the identification process for FS, TI, and IU, the system must satisfy the damping condition. This requires the coefficient of the fast variable, $\gamma_{2}$, to be positive and substantially larger than that of the slow variable, $\gamma_{1}$ (i.e., $\left|\gamma_{2}\right|\gg \left|\gamma_{1}\right|$). This condition is essential because it ensures that the fast variable decays rapidly enough to be “enslaved” or controlled by the order parameter, allowing the slow variable to dictate the system’s trajectory. Empirical results show that the motion equations for the FS-TI, TI-IU, and FS-IU systems all meet these criteria. Consequently, effective order parameters exist between every pair within the triad. These variables do not merely follow a linear, one-way “transmission chain,” where one factor passes influence to the next in a simple sequence, but instead engage in multi-directional feedback loops. This results in the system forming a synergy network, distinct from a single transmission chain, where the three components interact reciprocally to drive integrated development.

(2) Potential Function

To provide a rigorous comparative framework for the dynamic characteristics of the FS-TI, TI-IU, and FS-IU systems, the potential functions, stationary solutions, and stability conditions are derived from the order parameters identified in Table 3. These metrics are essential for mapping the energy landscape and evolutionary trajectories of each system, enabling a quantitative evaluation of their relative stability and phase-transition behaviors, as summarized in Table 4.

The stationary solution $q^{*}=0$ is of fundamental theoretical significance as it represents the initial undisturbed state of the system prior to structural evolution. Substituting $q^{*}=0$ into the potential functions yields second derivatives $V^{\prime \prime }$ of 0.0252, 0.0271, and 0.0445, all of which are positive. Initially, the potential function reaches its minimum at the origin, characterizing a stable equilibrium state. For *q* > 0, the critical threshold points are located at 0.45, 1.23, and 0.56, respectively, with symmetric distributions along the negative half-axis for *q* < 0. These numerical variations reveal distinct differences in system resilience: the TI-IU system, having the highest threshold (1.23), exhibits the greatest stability and resistance to transitions, whereas the FS-TI system (0.45) is the most sensitive to perturbations. The potential function achieves its maximum at these unstable critical points, which dictate the ultimate direction of the system’s evolution. Changes in the external environment or internal parameters trigger interactions between the state variable *q* and control parameters ($\gamma_{1}$, $a,\;{\;\gamma }_{2},\;\;b$). Such interactions modify the potential energy landscape, thereby disrupting the original equilibrium and propelling the system across the threshold toward a new state.

The FS-TI, FS-IU, and TI-IU systems represent the core interactive mechanisms within the regional economic framework, designed to evaluate how financial resources and technical progress synergistically drive structural transformation. This study utilizes potential functions to delineate the evolutionary paths and coordination mechanisms inherent in these coupled subsystems. By identifying critical thresholds, the analysis aims to reveal the energy barriers that must be overcome to achieve high-level systemic synergy.

For the FS-TI system, there is a significant interaction between financial support and technological innovation, with its minimum point located at (0, 0). The threshold point (0.45, 0.00127) represents the primary obstacle to higher-level coordination; when the synergistic effect approaches zero, the system resides at a stable equilibrium and surmounting the critical threshold of 0.45 necessitates external intervention. Once this boundary is crossed, the potential energy characteristics reflect a state of transformative cooperation. This system exhibits relative elasticity, where external shocks—such as targeted fiscal subsidies or shifts in credit policy—can perturb the dynamic equilibrium and lower the energy barrier required for the system to transition between coordination states.

The FS-IU system exhibits similar evolutionary characteristics to the other two systems. Substituting the stationary solution $q^{*}=0$ into the equation yields a second derivative of 0.0271 and a critical threshold of 0.56, indicating that the potential function curve shows a stable accumulation state near the origin. Overcoming this threshold requires the injection of external force. Once financial support exceeds this critical level, it potentially facilitates overcoming the path dependence of traditional industrial structures and promotes the development of the entire system towards more advanced industrial forms.

In the TI-IU system, a synergy mechanism is formed whereby changes in technological innovation lead to shifts in industrial upgrading, thereby affecting the evolution of the composite system. The potential energy curve shows steep features near the threshold points (1.23, 0.0170) and (−1.23, 0.0170). The symmetry between these positive and negative values signifies that the system is anchored in a deep potential well, where bidirectional deviations from the equilibrium point necessitate equivalent magnitudes of technological breakthrough, specifically exceeding the absolute critical value of 1.23 to initiate structural change. Compared with the FS-TI and FS-IU systems, the potential energy surface of the TI-IU system is steeper and the potential well at the origin is deeper, reflecting that industrial upgrading is more profoundly constrained by the prevailing technological level.

#### 4.4.2. Synergy Degree

**Figure 4:**
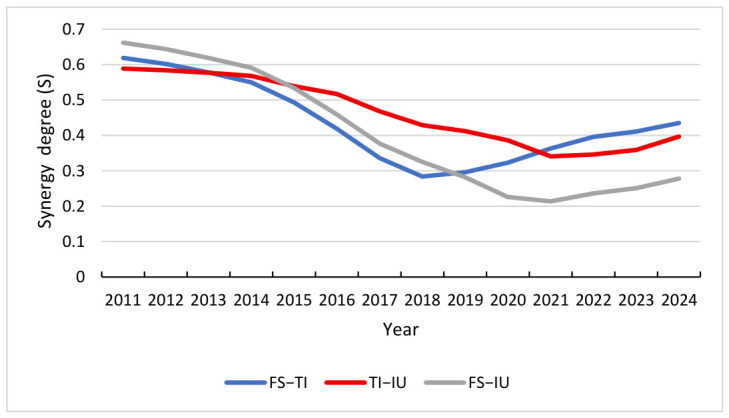
Synergy degree trend of FS-TI, TI-IU and FS-IU.

Potential function analysis uncovers the dynamic evolution mechanisms within the composite system. To evaluate the macro-performance and robustness, we conduct a comparative analysis of the FS-TI, TI-IU, and FS-IU subsystems. The synergy degree *S* is calculated based on the distance d from Equation (10), where a higher value of *S* signifies a higher level of synergy. The synergy degree results for each subsystem are presented in Figure 4 and Table 5.

#### 4.4.3. Synergy Effects of FS-TI, TI-IU and FS-IU

The trajectories of the synergy degrees offer a detailed view of the systemic shifts occurring over the study period. From Figure 4, it can be seen that the S-curves exhibit an asymmetric U-shaped feature, characterized by a marked early-stage decline, a bottom adjustment, and a slow rebound in the later stage. These characteristics are closely related to events such as the integration and adjustment of China’s financial market in 2015–2016, the Sino-US trade friction that began in 2018, and the COVID-19 pandemic that began in 2020. Specifically, the synergy of FS-TI has continued to decline from 0.619 in 2011, reaching a historical low of 0.284 in 2018. This evolutionary process can be divided into four stages: From 2011 to 2015, the synergistic effect remained relatively stable. From 2016 to 2018, it was a period of deep adjustment, with a precipitous decline in synergy, reflecting a serious structural mismatch between financial resource allocation and technological innovation demand. The period from 2019 to 2020 was a low adjustment period, with a low range fluctuation of synergy between 0.296 and 0.323, as the system reached a critical point in finding a new equilibrium. From 2021 to 2024, it entered a preliminary recovery period, and the synergy gradually recovered to 0.435. This indicates that after experiencing long-term systemic imbalances, compatibility is improving due to policy and market adjustments.

The S-curve of TI-IU system exhibits a continuous downward trajectory followed by a flattening phase, characterized by relatively smooth fluctuation rhythms. The system synergy index had an initial value of 0.589 in 2011 and underwent a decade-long decline, reaching a low of 0.341 in 2021. In contrast to the FS-TI system, the TI-IU system did not demonstrate a sharp “bottoming out” recovery but instead entered a period of low-level stability after 2021. This phenomenon suggests that the conversion of technological achievements into industrial productivity encounters formidable barriers and structural bottlenecks, specifically the misalignment between academic research outputs and actual market demand, as well as the high capital risks associated with the pilot-scale testing of core technologies. Although the current expansion of technological investment and the optimization of technology transfer policies are expected to gradually mitigate these constraints, the inherent complexity of industrial restructuring implies that the systems’ synergy is difficult to achieve significant cross-stage development in the short term, resulting in the observed characteristics of persistent low-level stability until 2024.

The S-curve of FS-IU system displays a pattern of a high initial value followed by a sharp and accelerating decline. The index recorded a relatively high level of 0.662 in 2011, representing the highest initial synergy among the three systems. However, a severe downward trend ensued, particularly between 2014 (0.591) and 2020 (0.226), eventually reaching an absolute bottom of 0.214 in 2021. This trajectory reflects an acute imbalance during the economic transition period between the traditional financial supply model and the requirements of modern industrial upgrading. Specifically, the traditional credit system’s heavy reliance on indirect financing and physical collateral fails to address the needs of modern industrial sectors, such as asset-light high-tech startups that require long-term “patient capital” rather than short-term loans. Although a slight rebound was observed after 2021, the overall synergy level remains depressed. This indicates that the financial-industrial support chain is particularly vulnerable to external environmental shocks, and the reconstruction of its synergy mechanism will require prolonged structural adjustment.

#### 4.4.4. Theil Index of FS-TI, TI-IU and FS-IU

**Figure 5:**
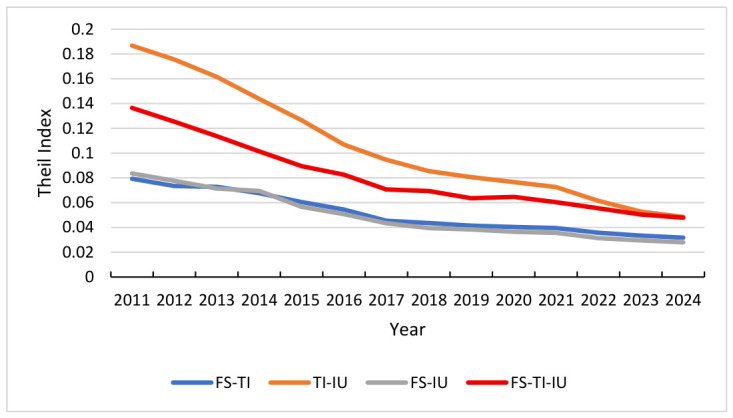
Theil index among the systems of FS-TI, FS-IU, and TI-IU.

Based on Equation (12), the Theil index was calculated to quantify development imbalances among the FS, TI, and IU subsystems, with results presented in Figure 5. Overall, the index demonstrates a significant downward fluctuation toward convergence, indicating a narrowing gap in the development scale and level of the three subsystems. This process exhibits distinct phased characteristics: an initial rapid convergence period where the FS-TI system addressed historical scale mismatches, followed by a stabilization phase where the index fluctuates within marginal intervals, reaching a relatively balanced equilibrium.

While these overarching trends suggest increasing systemic alignment, a more granular analysis reveals specific dynamics regarding the reduction in non-equilibrium conditions across subsystem pairings. Notably, the TI-IU system exhibits the most significant reduction in non-equilibrium conditions. However, the curve’s trajectory highlights a persistent gap between technological innovation investment and industrial output, suggesting that spillover effects have not been fully converted into industrial drivers. This bottleneck may stem from a lag in the commercialization of research and a mismatch between high-end innovation supply and existing industrial capacity; overcoming this requires strengthening technology transfer mechanisms and fostering deeper industry-university-research integration. Conversely, the difference coefficients for the FS-TI and FS-IU systems have remained consistently low, proving that financial supply, technology investment, and industrial demand have reached a high level of aggregate closeness. This sustained convergence reflects enhanced efficiency in financial resource allocation and a more robust innovation driving force.

## 5. Discussion

### 5.1. Analysis of the Relationship Between Synergy and Volatility

#### 5.1.1. Spearman Rank Correlation Coefficient

To statistically verify the inverse relationship between volatility and synergy, Spearman rank correlation coefficients were calculated. As shown in Table 6, the correlation coefficients for the FS-TI, TI-IU, and FS-IU subsystems are between −0.702 and −0.864, between −0.481 and −0.579, and between −0.368 and −0.645, respectively, and are statistically significant. These results confirm that higher volatility is significantly negatively correlated with synergy across all three subsystems. From a practical standpoint, these negative ranges indicate that as the intensity of fluctuations in financial support, technological innovation, or industrial upgrading increases, the stability and collaborative efficiency of the system diminish, thereby hindering the achievement of a high-synergy state.

#### 5.1.2. Relationship Between S and Δq

**Figure 6:**
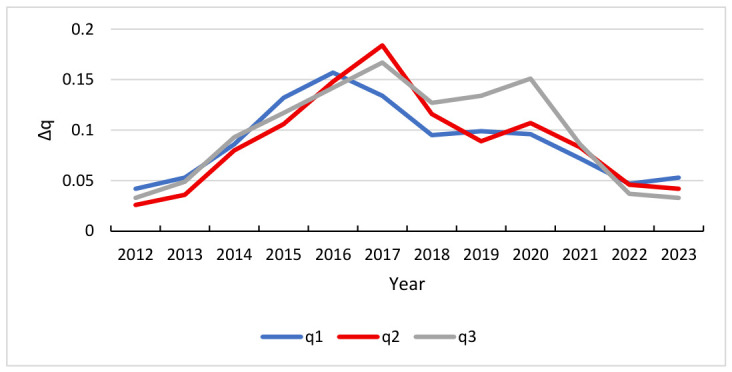
Trend chart of q value changes.

To complement these statistical findings, Figure 6 illustrates the fluctuation intensity (Δq) of these subsystems, offering a visual representation of the interaction between driving factors and synergy states. By mapping the instability within the evolutionary process, Figure 6 provides a grounded context for the negative correlations observed in Table 6, highlighting how specific periods of high volatility correspond to disruptions in the system’s overall synergistic development.

**Figure 7:**
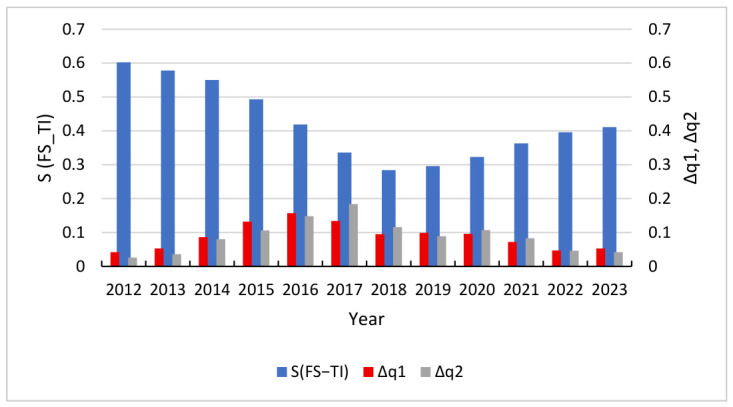
Trend chart of S(FS-TI) vs. Δq1, Δq2.

**Figure 8:**
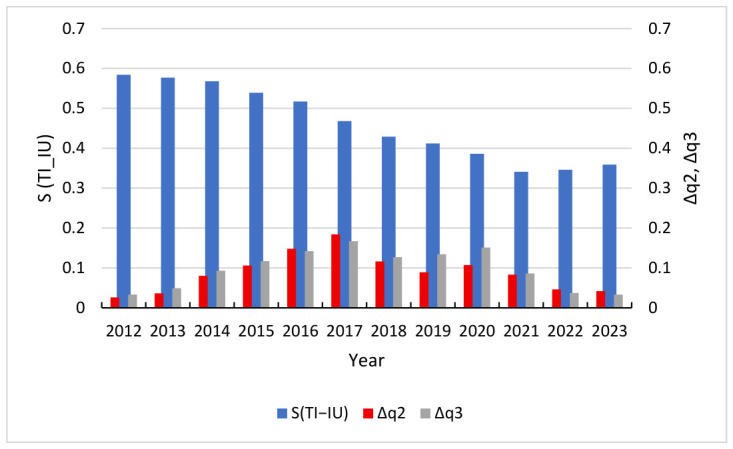
Trend chart of S(TI-IU) vs. Δq2, Δq3.

**Figure 9:**
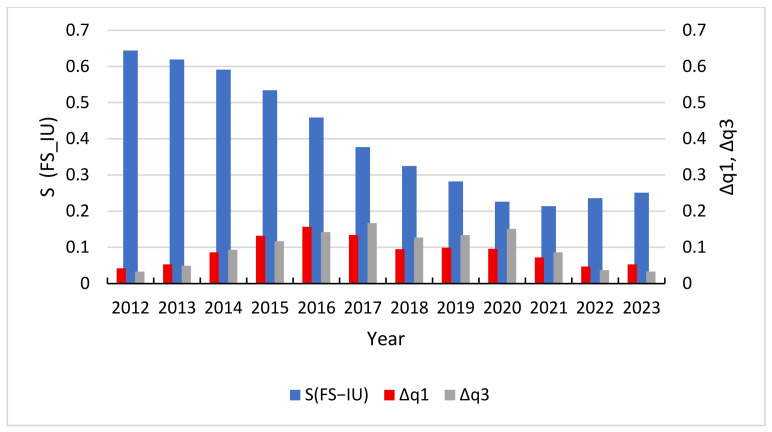
Trend chart of S(FS-IU) vs. Δq1, Δq3.

To effectively capture the dynamic interplay between subsystem stability and the overarching integration of the system, this study adopts a comparative visualization approach. Figure 7, Figure 8 and Figure 9 utilize a dual Y-axis configuration to map synergy and volatility onto a shared temporal scale, with the primary Y-axis representing the degree of synergy and the secondary Y-axis representing the volatility index. This methodological choice facilitates a direct examination of the co-movement between these variables. Analysis of the data reveals a pronounced inverse relationship between subsystem volatility and system-wide synergy, indicating a causal link where heightened instability disrupts the mechanisms of coevolution and undermines organizational order.

As shown in Figure 7, in the early stages of FS-TI evolution, when the volatility level was relatively low, the system’s synergy remained at a high level, reaching 0.602 in 2012. As the volatility level significantly increased and reached its peak around 2017, intense random fluctuations significantly affected the system’s orderliness. Due to the internal feedback of the system having a certain time delay, the synergy degree subsequently dropped to a historical low of 0.284 in 2018. This sharp numerical decline following the peak of instability confirms the logic of evolution, specifically that the accumulation of internal entropy from excessive fluctuations disrupts the self-organizing processes necessary for synergy. The greater the volatility level, the weaker the synergistic effect. When the volatility level is too high, the efficiency of financial capital’s support for technological innovation will be significantly disrupted, and the system will transition from an ordered state to a disordered state.

The inverse correlation observed in the FS-TI system is also empirically supported in both the TI-IU and FS-IU subsystems. As shown in Figure 8 and Figure 9, the volatility level of each subsystem showed a significant increase during the middle stage of evolution. This trend indicates that the system is in a high-risk structural oscillation period. This unstable state directly leads to a sustained decline in the level of synergy during this stage. Due to the continuous impact of external uncertainty, the synergy between TI-IU and FS-IU reached absolute lows of 0.341 and 0.214 in 2021, respectively. This indicates that when the volatility level exceeds the carrying capacity of the industrial structure, it can lead to serious failure of the synergy mechanism. With the gradual decline of volatility indicators in the later stages of evolution, the stability of subsystems has been enhanced; however, the level of synergy has only begun to show weak signs of recovery, with the synergy indices for both subsystems showing a noticeable upward inflection of approximately 0.056 to 0.064 units from their 2021 troughs by 2024.

### 5.2. Further Analysis

Empirical research reveals a positive and asymmetric U-shaped relationship among FS, TI, and IU, echoing the conclusions of He et al. (2024) [15]. Specifically, TI exerts a fundamental driving and synergistic influence on IU. However, the conversion of TI into IU is not an immediate process; instead, it necessitates extensive structural realignments in industrial production and resource allocation. Consequently, the synergy within the TI-IU system follows a downward trajectory before eventually stabilizing at a low-level equilibrium. These results align with the observation by Mensah et al. (2023) [8] that TI requires significant resource reallocation and suggest that converging development levels alone does not guarantee enhanced synergy.

Similarly to the coupling coordination analysis by Wang and Dong (2024) [37], our findings indicate that FS bolsters corporate TI capabilities by providing vital R&D funding. Nevertheless, the efficacy of this support is contingent upon the precise alignment between financial provision and the specific multidimensional requirements of TI and IU. Our model results demonstrate that when FS fails to match these needs or is disrupted by external volatility, its effectiveness diminishes, leading to a reduction in synergy. This phenomenon is also noted in the studies of Meng et al. (2022) [14] and Bu et al. (2025) [16].

To validate the reliability of the Haken model, its results were compared with the Coupling Coordination model applied by Nie et al. (2024) [35]. This comparison reveals that the FS-TI synergy derived from the Haken model follows a pattern parallel to the coupling degree identified in the reference model. Both metrics exhibit an asymmetrical U-shaped evolution trajectory, with synergy and coupling values remaining consistently within the medium-to-low range.

Based on the Haken model testing and the analysis of the Theil index, the results demonstrate a robust synergistic evolution among the FS-TI-IU subsystems. By identifying the dominant order parameters and confirming their collective self-organization through positive feedback loops, this research provides empirical evidence that these components act in a coordinated manner to drive system optimization, thus validating Hypothesis 1.

While few systematic studies have explored the constraining effect of volatility on synergy, this research identifies a significant negative correlation between the amplitude of fluctuations and the level of systemic synergy. Empirical analysis reveals that excessive random fluctuations disrupt the precise synchronization of policy signals and resource flows between subsystems, thereby weakening the feedback mechanisms essential for maintaining structural stability. As the quantitative data shows a marked decline in the system’s internal orderliness when these fluctuations exceed critical thresholds, Hypothesis 2 is substantiated.

## 6. Conclusions

This study empirically analyzed the synergistic effects of financial support, technological innovation, and industrial upgrading based on the Haken model. The research results indicate that the interaction among these subsystems forms a composite synergic mechanism rather than a single linear conduction chain. Specifically, FS provides the necessary capital for TI, which, in turn, facilitates IU through technological spillover, while IU generates new market demand for specialized financial services, creating a self-reinforcing feedback loop that promotes mutual development.

The synergistic effect exhibits significant fluctuations over time. While the subsystems remained synchronized in their response to structural adjustments, the system experienced severe disruptions in 2018 and 2021. The 2018 Sino-US trade friction and the 2021 global supply chain instabilities triggered by the COVID-19 pandemic hindered capital flows and technological dissemination, thereby weakening systemic coordination. However, under the guidance of macro policies and industrial restructuring, the system has shown a resilient rebound. Although currently in a low-level adaptation phase, the internal linkage mechanism remains active and is evolving toward orderliness through self-regulation.

The system’s synergy is currently at a medium-to-low level and is strongly constrained by volatility, with a significant inverse relationship between the two. High volatility often leads to a “low-level fluctuation trap”—a state where excessive uncertainty prevents the system from forming stable structural bonds—thereby decreasing internal orderliness. Conversely, when volatility is moderated, the system can more effectively repair internal imbalances, cross “development thresholds”—the critical transition points required to reach higher organizational maturity—and move toward superior synergetic states.

Given this inverse relationship, macroeconomic policies should prioritize the precise regulation of volatility within each subsystem. By optimizing industrial structures to mitigate the negative impacts of uncertainty, policymakers can ensure the deep integration of financial support, technological innovation, and industrial upgrading. The policy orientation should shift from simple scale expansion to synergistic efficiency, guiding the system to break free from current low-level bottlenecks and ultimately evolve toward a high-quality, stable synergetic state.

## Figures and Tables

**Table 1:** Evaluation criteria for synergy level.

Degree of Synergy	Level of Synergy
0 < *S* ≤ 0.4	Low-level synergy
0.4 < *S* ≤ 0.7	Moderate synergy
0.7 < *S* ≤ 1.0	High-level synergy

**Table 2:** Indicator weight allocation.

Synergetic System Elements	Measurement Indicators	Weight
Financial Support (FS)	X_11_: Increase in revenue of the financial industry	0.3112
	X_12_: Loan amount for high-tech and industrial upgrading	0.2241
	X_13_: Transaction amount of electronic banking business	0.3318
	X_14_: Green credit quota	0.1329
Technological Innovation (TI)	X_21_: Full-time equivalent of R&D personnel	0.1548
	X_22_: R&D investment in high-tech products	0.3681
	X_23_: The number of authorized patents	0.1985
	X_24_: Sales revenue from high-tech products	0.2786
Industrial Upgrading (IU)	X_31_: The proportion of employment in the tertiary industry	0.2202
	X_32_: Ratio of tertiary added value to secondary industry	0.3443
	X_33_: Industrial structure hierarchy index	0.2392
	X_34_: Unit GDP carbon emission reduction	0.1963

**Table 3:** Motion equations of Haken model.

**System**	Motion Equation	Equation Parameters	Statistical Test
*1. q1 = FS* *q2 = TI*	$q_{1}\left(t\right)=0.9748q_{1}\left(t-1\right)+0.1692q_{1}\left(t-1\right)q_{2}\left(t-1\right)$ $q_{2}\left(t\right)=0.8809q_{2}\left(t-1\right)+0.0881q_{1}^{2}\left(t-1\right)$	$\gamma_{1}=0.0252;$	$F_{1}=6.12\;***;$
		a=−0.1692;	$P_{1}<0.001;$
		$\gamma_{2}=0.1191$;	$F_{2}=7.13\;***;$
		b=0.0881	$P_{2}<0.001$
*2. q1 = FS* *q3 = IU*	$q_{1}\left(t\right)=0.9729q_{1}\left(t-1\right)+0.1481q_{1}\left(t-1\right)q_{3}\left(t-1\right)$ $q_{3}\left(t\right)=0.8905q_{3}\left(t-1\right)+0.0638q_{1}^{2}\left(t-1\right)$	$\gamma_{1}=0.0271;$	$F_{1}=11.56\;***;$
		a=−0.1481;	$P_{1}<0.001;$
		$\gamma_{2}=0.1095$;	$F_{2}=31.12\;***;$
		b=0.0638	$P_{2}<0.001$
*3. q2 = TI* *q3 = IU*	$q_{2}\left(t\right)=0.9555q_{2}\left(t-1\right)+0.0436q_{2}\left(t-1\right)q_{3}\left(t-1\right)$ $q_{3}\left(t\right)=0.8568q_{3}\left(t-1\right)+0.0961q_{2}^{2}\left(t-1\right)$	$\gamma_{1}=0.0445$;	$F_{1}=2.63\;*;$
		a=−0.0436;	$P_{1}=0.032\;**;$
		$\gamma_{2}=0.1432;$	$F_{2}=13.89\;***;$
		b=0.0961	$P_{2}<0.001$

Note: *, **, and *** indicate significance at the 10%, 5%, and 1% confidence levels, respectively.

**Table 4:** Results of potential functions for FS-TI, TI-IU and FS-IU.

Subsystem	Potential Function V(q)	Stationary Solutions	Second Derivative $V^{\prime \prime }$
FS-TI	$V\left(q_{1}\right)=0.0126q_{1}^{2}-0.0313q_{1}^{4}$	${q_{1}}^{*}=0$, ${q_{1}}^{**}$ ≈ 0.45, ${q_{1}}^{***}\approx -0.45$	$V^{\prime \prime }=0.0252-0.3755q_{1}^{2}$
FS-IU	$V\left(q_{2}\right)=0.0136q_{2}^{2}-0.0216q_{2}^{4}$	${q_{2}}^{*}=0$, ${q_{2}}^{**}\approx 0.56,$ ${q_{2}}^{***}\approx -0.56$	$V^{\prime \prime }=0.0271-0.2589q_{2}^{2}$
TI-IU	$V(q_{3})=0.0223q_{3}^{2}-0.0073q_{3}^{4}$	${q_{3}}^{*}=0$ ${q_{3}}^{**}$ ≈ 1.23, ${q_{3}}^{***}\approx -1.23$	$V^{\prime \prime }=0.0445-0.0878q_{3}^{2}$

**Table 5:** Synergy degree of FS-TI, TI-IU and FS-IU.

Year	d_FS−TI_	S_FS−TI_	d_TI−IU_	S_TI−IU_	d_FS−IU_	S_FS−IU_
2011	0.381	0.619	0.411	0.589	0.338	0.662
2012	0.398	0.602	0.416	0.584	0.356	0.644
2013	0.422	0.578	0.423	0.577	0.381	0.619
2014	0.450	0.55	0.432	0.568	0.409	0.591
2015	0.507	0.493	0.461	0.539	0.466	0.534
2016	0.581	0.419	0.483	0.517	0.541	0.459
2017	0.664	0.336	0.532	0.468	0.623	0.377
2018	0.716	0.284	0.571	0.429	0.675	0.325
2019	0.704	0.296	0.588	0.412	0.718	0.282
2020	0.677	0.323	0.614	0.386	0.774	0.226
2021	0.637	0.363	0.659	0.341	0.786	0.214
2022	0.604	0.396	0.654	0.346	0.764	0.236
2023	0.589	0.411	0.641	0.359	0.749	0.251
2024	0.565	0.435	0.603	0.397	0.722	0.278

**Table 6:** Spearman rank correlations of FS-TI, TI-IU and FS-IU.

Subsystem	Variable	Spearman Rank Correlation Coefficient	*p*
FS-TI	S_FS−TI_ vs. Δq1	−0.702 ***	0.0001
	S_FS−TI_ vs. Δq2	−0.864 ***	0.0001
TI-IU	S_TI-IU_ vs. Δq2	−0.481 ***	0.0006
	S_TI-IU_ vs. Δq3	−0.579 ***	0.0001
FS-IU	S_FS-IU_ vs. Δq1	−0.368 **	0.0105
	S_FS-IU_ vs. Δq3	−0.645 ***	0.0001

Note: **, and *** indicate significance at 5%, and 1% confidence levels, respectively.

## Data Availability

The data presented in this study are available on request from the corresponding author.
